# Harnessing positive species interactions as a tool against climate-driven loss of coastal biodiversity

**DOI:** 10.1371/journal.pbio.2006852

**Published:** 2018-09-04

**Authors:** Fabio Bulleri, Britas Klemens Eriksson, Ana Queirós, Laura Airoldi, Francisco Arenas, Christos Arvanitidis, Tjeerd J. Bouma, Tasman P. Crowe, Dominique Davoult, Katell Guizien, Ljiljana Iveša, Stuart R. Jenkins, Richard Michalet, Celia Olabarria, Gabriele Procaccini, Ester A. Serrão, Martin Wahl, Lisandro Benedetti-Cecchi

**Affiliations:** 1 Dipartimento di Biologia, Università di Pisa, CoNISMa, Pisa, Italy; 2 Groningen Institute for Evolutionary Life Sciences, University of Groningen, Groningen, the Netherlands; 3 Plymouth Marine Laboratory, Plymouth, United Kingdom; 4 Dipartimento di Scienze Biologiche, Geologiche ed Ambientali, University of Bologna, CoNISMa, Ravenna, Italy; 5 CIIMAR-Interdisciplinary Center of Marine and Environmental Research, Matosinhos, Portugal; 6 Institute of Marine Biology, Biotechnology and Aquaculture, Hellenic Centre for Marine Research, Thalassokosmos, Crete, Greece; 7 NIOZ Royal Netherlands Institute for Sea Research, Department of Estuarine and Delta Systems and Utrecht University, Yerseke, the Netherlands; 8 Earth Institute and School of Biology and Environmental Science, University College Dublin, Dublin, Ireland; 9 Sorbonne Université, CNRS, UMR 7144 Adaptation et Diversité en Milieu Marin, Roscoff, France; 10 Sorbonne Université, CNRS, Laboratoire d'Ecogéochimie des Environnements Benthiques (LECOB), Banyuls-sur-Mer, France; 11 Ruđer Bošković Institute, Center for Marine Research, Rovinj, Croatia; 12 School of Ocean Sciences, Bangor University, Anglesey, United Kingdom; 13 UMR CNRS 5805 EPOC, University of Bordeaux, Talence, France; 14 Departamento de Ecoloxía e Bioloxía Animal, Facultade de Ciencias del Mar, Campus Lagoas-Marcosende, Universidade de Vigo, Vigo, Spain; 15 Stazione Zoologica Anton Dohrn, Villa Comunale, Napoli, Italy; 16 CCMAR, CIMAR, University of Algarve, Campus de Gambelas, Faro, Portugal; 17 GEOMAR Helmholtz Centre for Ocean Research, Kiel, Germany

## Abstract

Habitat-forming species sustain biodiversity and ecosystem functioning in harsh environments through the amelioration of physical stress. Nonetheless, their role in shaping patterns of species distribution under future climate scenarios is generally overlooked. Focusing on coastal systems, we assess how habitat-forming species can influence the ability of stress-sensitive species to exhibit plastic responses, adapt to novel environmental conditions, or track suitable climates. Here, we argue that habitat-former populations could be managed as a nature-based solution against climate-driven loss of biodiversity. Drawing from different ecological and biological disciplines, we identify a series of actions to sustain the resilience of marine habitat-forming species to climate change, as well as their effectiveness and reliability in rescuing stress-sensitive species from increasingly adverse environmental conditions.

## Positive species interactions under climate change

Anthropogenic climate change is causing unprecedented alterations to Earth’s ecosystems [1,2]. Modifications in species distribution and abundance as a consequence of altered environmental conditions can be the direct result of physiological and/or phenological responses [3]. More often, climate-induced modifications in individual physiology, phenology, and behavior scale up to the community level through the filter of species interactions [4]. Nonetheless, species interactions are still seldom incorporated into models aiming to forecast species distribution under future climate scenarios [5,6].

Although terrestrial and marine studies have started addressing the effects of climate change on the balance between negative and positive species interactions (Box 1) [7–11], the role of habitat-formers (Box 1) in shaping future patterns of species distribution is yet to be fully explored. This is at odds with compelling evidence showing that habitat-formers frequently facilitate other species in otherwise hostile environments [5,8,12–15] and can enhance conservation and restoration success [16–18]. Habitat-formers have allowed species to persist under dramatic changes in climate in the past and acted as important evolutionary forces. For instance, environmental stress amelioration by canopy-forming Quaternary plants has allowed Tertiary plant lineages adapted to moist conditions to persist despite the onset of an unfavorable climate [19]. Indeed, biogenic modification of abiotic conditions (Box 1) underpins pivotal chapters in the evolution of life on Earth; in the Cambrian Period, the development of biomineralised skeletons (e.g., trilobites and other arthropods), a response to the advent of predation, caused reworking and oxygenation of ocean sediments (i.e., the burrowing revolution), giving rise to the ancestors of many modern groups of animals [20]. Milder conditions due to warming may reduce the reliance of extant species on habitat-formers in some extreme environments, such as alpine and arctic tundra [7]. There is, however, indisputable evidence that increasingly harsher physical conditions are a major driver of the current biodiversity crisis across ecosystems on Earth [1,2], suggesting that the importance of physical stress amelioration by habitat-formers is set to increase under future climate scenarios.

**Box 1. Glossary**

**Positive species interactions**: Interactions among species, also referred to as facilitative interactions or facilitation, in which at least one of the participants benefits from the presence of the other, while neither is disadvantaged. These include interactions between coevolved, mutually obligate organisms as well as looser, facultative interactions between species that did not coevolve.

**Habitat-former**: A species able to support the persistence of other species by providing suitable environmental conditions, enhancing the availability of or access to limiting resources, or reducing the effects of negative species interactions, such as competition, predation, and diseases. Habitat-formers include ecosystem engineers, which are defined as organisms that affect other species through the creation, modification, and maintenance of habitat. Biotic and abiotic conditions are not necessarily optimal (relative to other habitats) for all the species found in the presence of a habitat-former.

**Biogenic modification of environmental conditions**: Modification of environmental conditions operated by a living organism (i.e., a habitat-former). Similarly, biogenic amelioration or buffering of environmental stress refers to the case in which the presence of a living organism reduces the intensity of stressful environmental conditions for other species.

**Biogenic refugia**: Habitats formed by living organisms and of limited spatial extent that allow other species to escape adverse environmental or biological conditions and from which they can subsequently expand when suitability of external conditions is restored.

**Benefactor and beneficiary species**: The benefactor is a species able to deliver benefits to other species, defined as beneficiary species. A species may behave as a benefactor under some environmental conditions or resource availability levels but not under others. For example, an intertidal canopy-forming macroalga (i.e., the benefactor) can benefit understory species (i.e., the beneficiaries), reducing heat and desiccation at high-shore levels. By contrast, it can negatively influence understory species lower on the shore, where heat and desiccation stress are less severe.

**Epigenetic mechanisms**: Mechanisms that form the basis of the dynamic regulation of gene expression through chromatin remodeling, DNA methylation, noncoding RNA-associated genes, and histone modification. Epigenetic changes can be inherited but do not involve changes in the underlying DNA sequence.

**Assortative mating**: Nonrandom mating model in which the frequency of mating between individuals with a similar genotype and/or phenotype is higher than that expected by chance.

**Climate rescuer**: A habitat-former resistant/resilient to climate change providing suitable environmental conditions to species that would otherwise be unable to maintain viable populations under future climate scenarios.

**Fig 1:**
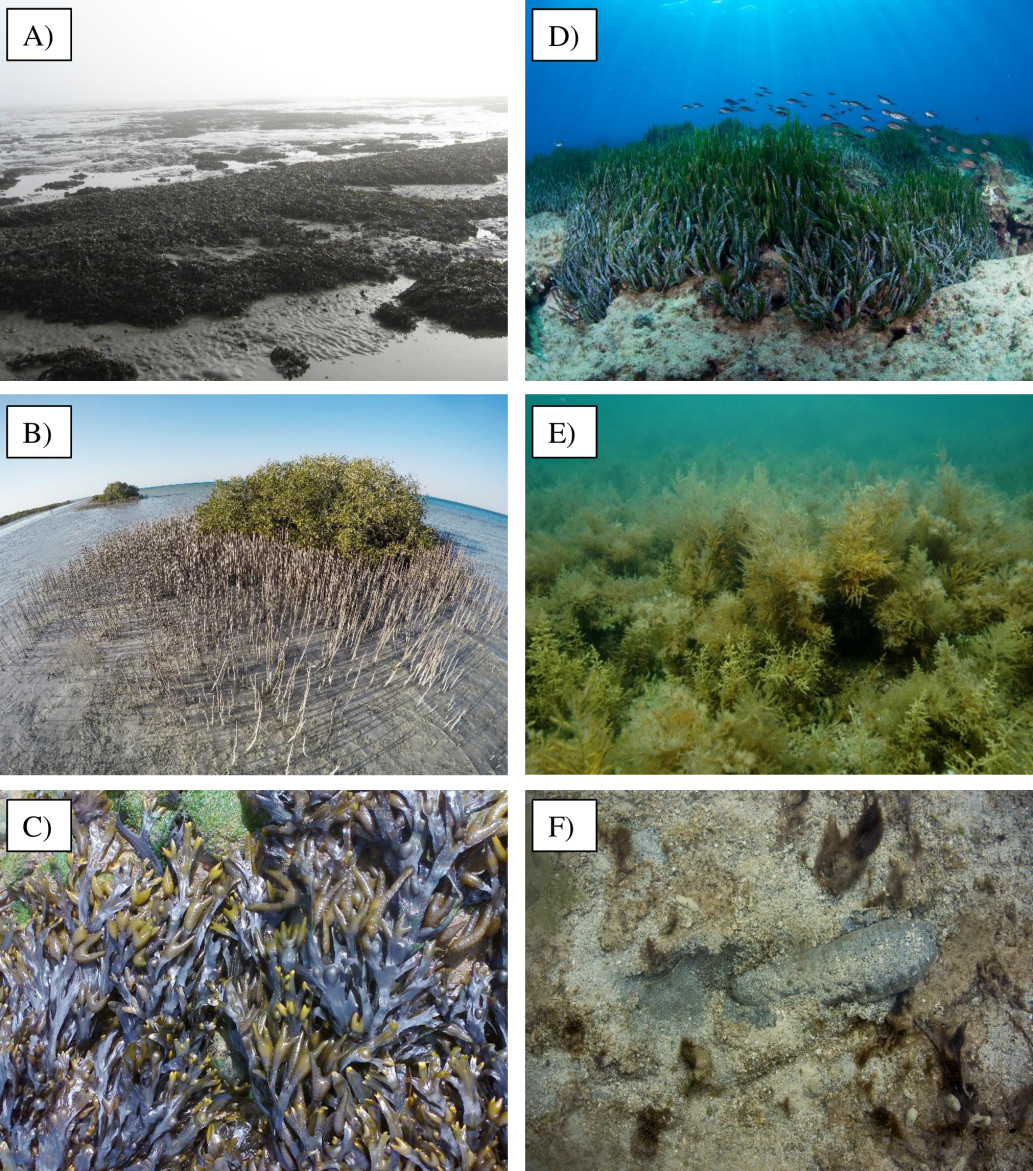
Habitat-formers in intertidal and subtidal environments. (A) Clumps of the mussel *Mytilus edulis* on a tidal flat in the Wadden Sea, the Netherlands (*Photo credit*: *B*.*K*.*E*. *Eriksson*); (B) mangrove trees of the species *Avicennia marina* along the central coasts of the Red Sea (*Photo credit*: *T*. *Dailianis*); (C) fronds of the brown seaweed *Fucus vesiculosus* at low tide on a rocky shore of the Iberian Peninsula *(Photo credit*: *E*. *Serrão*); (D) the seagrass *Posidonia oceanica* in shallow waters of Crete in the Aegean Sea (*Photo credit*: *T*. *Dailianis*); (E) multi-specific canopy stands formed by the brown seaweeds *Cystoseira barbata*, *C*. *compressa*, and *C*. *crinita* on shallow rocky reefs of Croatia in the northeast Adriatic Sea (*Photo credit*: *L*. *Iveša*); (F) burrowing by the sea cucumber *Holothuria scabra* exposes anoxic sediments on a reef flat in Fiji (*Photo credit*: *S*. *Lee*).

Habitat-formers are key in shaping community structure and ecosystem functioning in marine environments through both local and long-distance positive interactions that extend across coastal landscapes [12,13,21]. In transitional and shallow-water environments, the habitat-former concept has traditionally been applied to sessile species, such as mangroves, salt-marsh plants, seagrasses, macroalgae, bivalves, and corals [22] (Fig 1A–1E). However, mobile species that modify the characteristics of sediments through their burrowing or feeding activity (i.e., bioturbators, Fig 1F), such as holothurians, crustaceans, and polychaetes, could play a similar role from tidal flats to abyssal plains [23]. Here, we assess the circumstances under which biogenic amelioration of environmental stress may sustain coastal biodiversity and ecosystem functioning in the face of climate change and, hence, be used as a nature-based solution for coastal conservation and restoration.

## Biogenic refugia against climate change

Biogenic buffering of environmental stress has been documented in harsh, transitional habitats, such as intertidal rocky and sandy shores, mudflats, and salt marshes [12]. For example, intertidal macroalgal canopies or mussels beds reduce heat and desiccation stress during emersion, sustaining diversity and productivity of benthic communities [24,25]. However, while the role of geomorphological refugia for species persistence in the face of past and current changes in climate is recognized [26], that of biogenic refugia (Box 1) remains unexplored. Benefactors (Box 1) may provide climatically suitable habitat for stress-susceptible species, increasing their survival during acute climate-driven disturbance events, such as heatwaves or sea storms. For example, intertidal mussel clumps enhance cordgrass survival during severe drought events and function as nuclei for vegetative recovery in the aftermath [11]. In subtidal environments, macrophyte photosynthetic activity buffers calcifying organisms from ocean acidification by increasing pH [27,28]. Daily uptake of carbon dioxide (CO_2_) by plants increases pH within the surrounding diffusive boundary layer, and these effects can scale up to adjacent habitats, such as stony corals or mussel beds [28,29]. Subtidal canopies also attenuate wave action and, at shallow depths, light stress [30,31]. Below the sediment surface, biogenic activity can reduce the impacts of seasonal hypoxia driven by heatwaves. Seawater flushing and particle mixing by large burrowing marine invertebrates (i.e., bioturbation and bioirrigation) facilitate oxygenation of sedimentary pore water spaces and the burial of organic matter, ameliorating biogeochemical conditions within sediments [32,33]. Indeed, reduction of physical stress by bioturbators (e.g., temperature-driven hypoxia) may explain why the proportion of benthic species on soft sediments shifting their trailing edge at the pace predicted by seawater warming rates is lower than expected [34].

Facilitation can expand the distribution of beneficiary species beyond the range predicted from their physiological tolerance matrices [35–37]. The magnitude of the biogenic reduction of thermal stress may exceed—by far—the increment expected under warming climates. For example, intertidal canopies of the seaweed *Ascophyllum nodosum* reduced summer maximum rock temperatures in New England by as much as approximately 8 °C [24], and mussels and algal turfs ameliorated lethal and sublethal thermal stress over 14° of latitude [35].

Reliance of beneficiaries on biogenic amelioration of environmental conditions may increase under future climates, at least until beneficiary species possibly adapt to the new conditions. Thus, a large proportion of species in a community might become obligate associates with habitat-formers. The survival of beneficiary species would depend, first, upon the spatial and temporal extent of the biogenic refugia and, second, their fitness therein. Refugia might be too small to allow beneficiaries to maintain viable populations. In addition, life in biogenic habitats can entail costs due to competition either with the benefactor itself or other associated species [37].

## Adapt, move, or perish: The role of biogenic habitat

A species that is currently neither resistant (unaffected) nor resilient (able to recover) to climate change must either adapt or move to persist. Can habitat-formers influence the mechanisms underpinning species potential to i) exhibit plastic responses, ii) genetically adapt to novel environmental conditions, or iii) track suitable climates?

**Fig 2:**
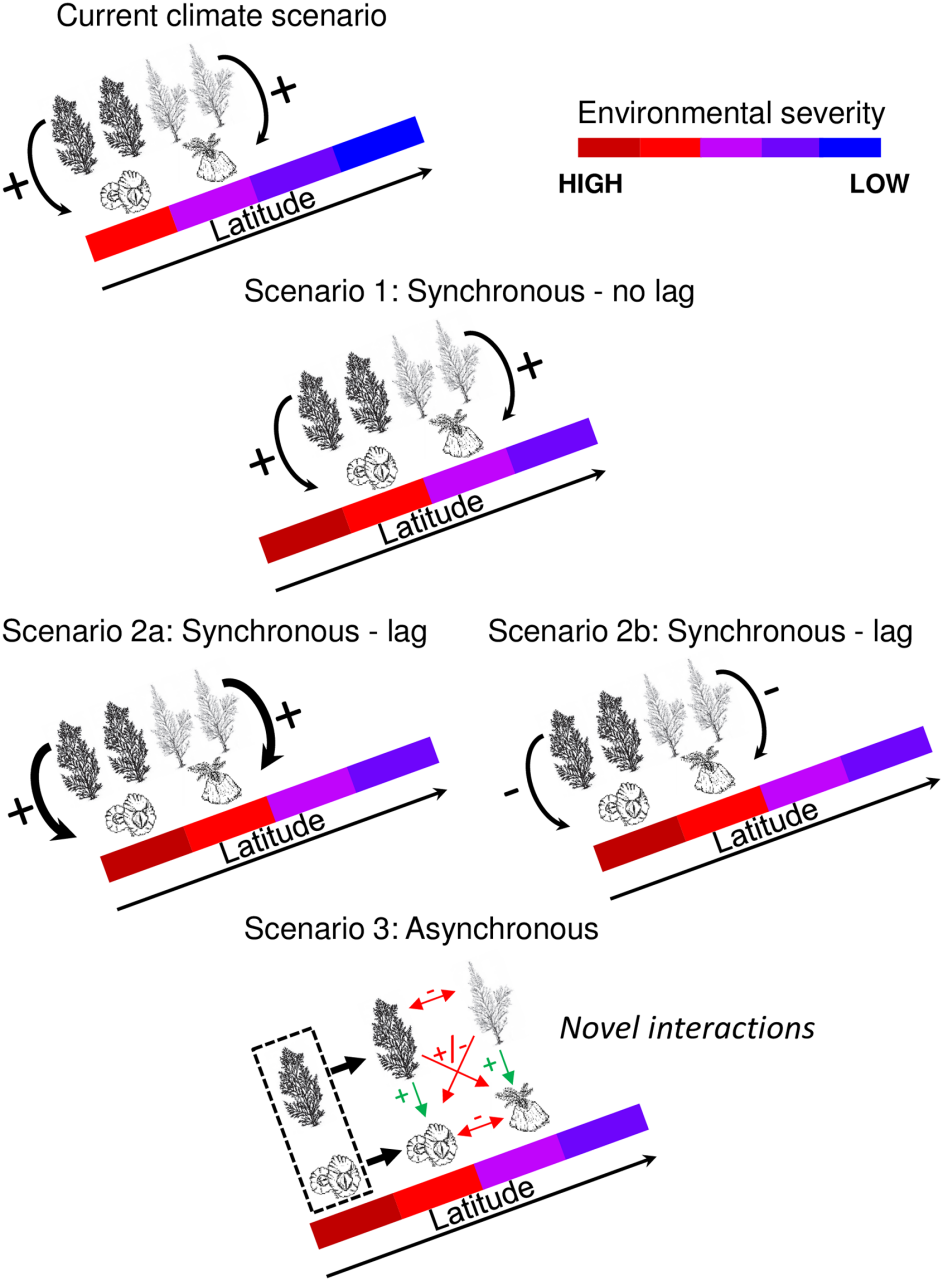
Alternative scenarios of interaction between benefactor and beneficiary species after climate change. Under the current climate, southern and northern canopy-forming macroalgae facilitate different species of barnacles (in the northern hemisphere, in this example). Under scenario 1, species migrate synchronously to track a suitable climate, resulting in no significant modifications of the interacting environment and no generation of novel interactions; extant positive interactions are maintained. Under scenario 2, all species exhibit the same time lag in migration and interact in harsher environmental conditions, resulting in either (a) a strengthening of positive interactions or (b), in the case in which levels of stress become excessive, in the collapse of facilitation. Under scenario 3, species migration is asynchronous, generating novel interactions. In this example, southern species migrate poleward and start interacting with extant, nonmigrating species. Positive interactions between each original pair of canopy-forming macroalgae and barnacles are likely to be maintained (green arrows). Novel interactions (red arrows) between canopy-formers and barnacles can be either positive or negative, while novel interactions between canopy-formers and between barnacles are likely to be negative.

i) Pre-existing phenotypic plasticity, allowing individuals to acclimate, may sustain short-term population persistence before evolutionary adaptation can take place [3]. Rapid adaptation to novel environmental conditions through the activation of alternative metabolic pathways or the modification of gene expression levels by epigenetic mechanisms (Box 1) has been demonstrated in marine organisms [38,39]. Acclimation can also influence subsequent generations, and biogenic habitats may facilitate species acclimation via developmental or transgenerational plasticity exposing individuals to sublethal temperatures during extreme events, such as heatwaves [40, 41].
ii) Adaptation to changing climate by selection of individual traits across generations can require time, especially for long-lived organisms. Body mass, reproduction type (e.g., sexual versus vegetative), and generation time influence local adaptation rates [42]. By virtue of their smaller body mass and shorter generation times, adaptation can be expected to be generally more rapid in beneficiary species than in habitat-formers. However, given that current climate-driven changes may modify marine habitats at rates fast exceeding the potential for adaptive, genetic change within populations, habitat-formers may buy population persistence time for stress-sensitive species. The evolutionary potential of positive interactions remains unquantified [43], but small-scale variation in the intensity of negative biotic interactions (e.g., predation) has been shown to promote rapid adaptive differentiation [44].
  Several lines of argument do suggest that biogenic habitats may influence fine-scale genetic structures of associated species. First, at the seascape scale, patches of habitat-formers alternating with open surfaces—a common configuration of transitional coastal environments—increase spatial heterogeneity in selective pressures, thus sustaining genetic polymorphism. This may explain the inverted dominance of two alleles found homozygous in barnacles living in exposed sites versus underneath a canopy-forming macroalga [45]. Second, enhanced aggregation of individuals seeking shelter in biogenic habitats, in association with limited dispersal and occurrence of within-habitat environmental gradients [10], can influence genetic structuring through isolation by distance [46]. Third, habitat-formers can elicit phenotypic variations in beneficiary species that, when involving reproductive traits, may enhance fine-scale genetic structuring through assortative mating (Box 1) [46,47]. Biogenic enhancement of genetic variation would be particularly important in populations at range edges since they may have lower genetic variability compared to central populations [48].
iii) Under lethal climate-driven stress, the synchrony of migration capacities determines species interaction outcomes at the leading edge of range shifts. Three different scenarios describe how climate change can alter species interactions [49]. In the first, all the species within a community migrate synchronously to track climate change without noteworthy modification of the interaction environment. Thus, facilitative effects of habitat-formers could be maintained in newly colonized areas (Fig 2, scenario 1).

In the second scenario, all species exhibit the same migration lag, thus interacting under changing environmental conditions. Enhanced levels of environmental stress may increase the frequency and/or intensity of positive interactions [15] (Fig 2, scenario 2a). For example, along the east coast of the United States, intertidal macroalgal canopies fostered cirriped survival at thermally stressful southern sites [24]. By contrast, at northern cooler sites, benefits were overridden by increased whelk predation. Progressive warming may strengthen stress mitigation benefits, shifting the net effect of canopies from negative to positive also at northern sites. Alternatively, facilitation may collapse if environmental stress becomes extreme and impairs the ability of the benefactor to deliver benefits [50] (Fig 2, scenario 2b).

In the third scenario, some species migrate toward cooler climates and start interacting with resident, nonmigrating species (Fig 2, scenario 3). Such novel interactions can be either positive or negative. Recruitment through seeds, spores, or larvae represents a critical stage of range shifts. Juvenile stages are often less tolerant to stressful conditions than adults, and biogenic stress amelioration might be crucial to enable their recruitment outside their current distributional range. For instance, on the east coast of the United States, salt marsh vegetation facilitates recruitment of the black mangrove *Avicennia germinans* at its northward distributional limit [51]. Positive effects do not necessarily stem from environmental stress reduction but might be generated by alleviation of resource limitation, competition, or predation pressure. For example, reefs formed by the Pacific oyster north of its former range provide native mussels with shelter from crab predation [52].

## What makes a habitat-former a climate rescuer species?

### Ecosystem-wide effects of environmental stress buffering

**Fig 3:**
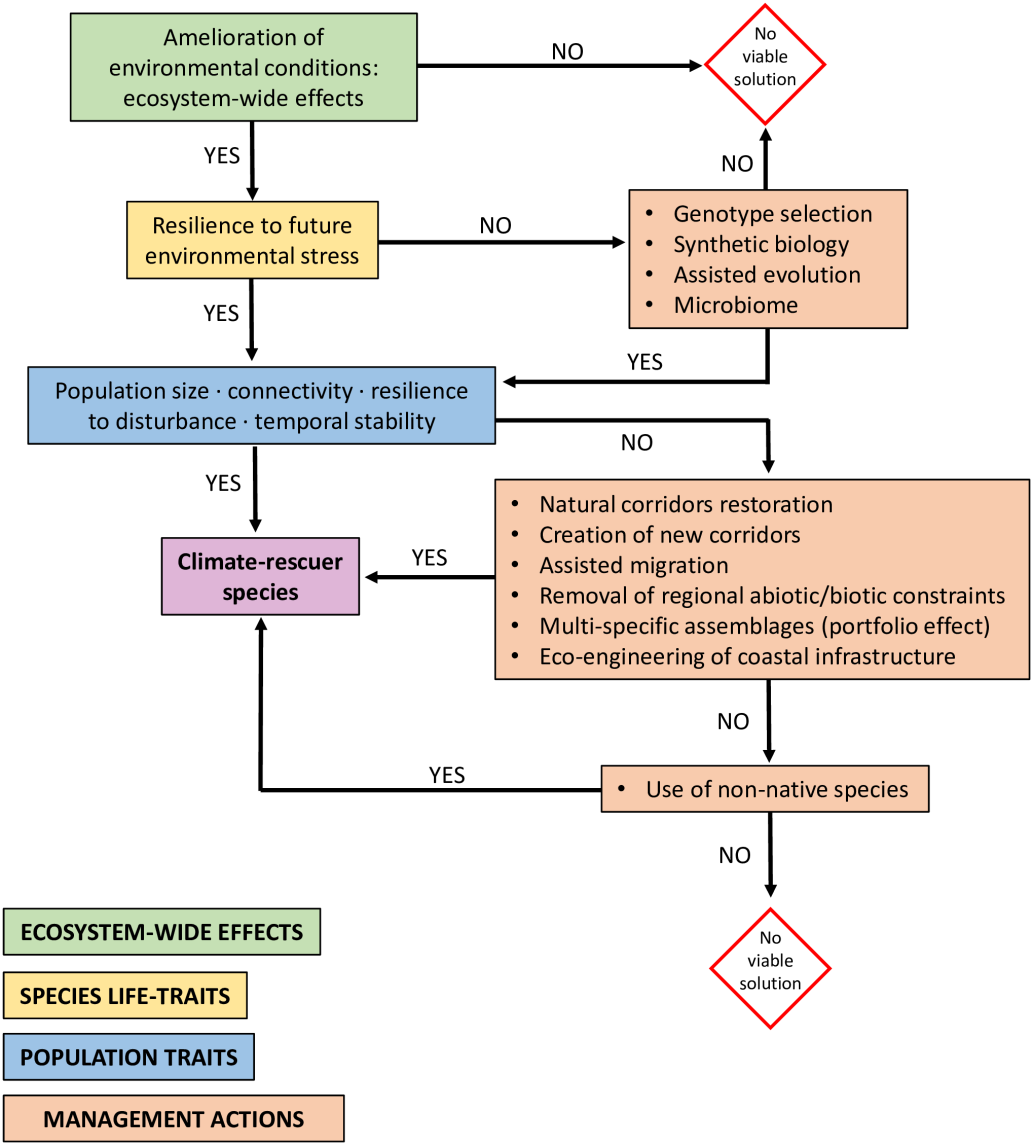
Climate rescuer identification and management. The diagram describes sequential steps toward the identification of a climate rescuer species and possible management actions aimed to sustain i) life traits underpinning its resistance to future environmental stress and ii) population traits that determine the strength and reliability of its positive effects on stress-sensitive species.

The first requisite of a climate rescuer (Box 1) is the ability to sustain biodiversity and ecosystem functioning through stress alleviation (Fig 3). This effect is not limited to temperature or desiccation but extends to other climate-related stressors, such as ocean acidification, hypoxia, increased UV radiation, and changing hydrodynamic regimes. Ideally, positive effects should not be limited to single habitats but should propagate to other ecosystems. Primary habitat-formers can provide substrates for other habitat-formers (facilitation cascades: [53]) or promote other species across the landscape (habitat cascades: [54]) through long-distance interactions [55]. Within this context, stress-tolerant species that facilitate other species both within and across habitats should be considered standout climate rescuers.

### Resilient morphology and phenology under changing climates

Climate rescuer species should be able to persist in increasingly stressful environments without facing morphological or phenological modifications that undermine their facilitative functionality (Fig 3). Climate rescuing would be supported if the benefactor can withstand a greater magnitude of change in a given climate-driven stressor than its beneficiaries whilst still sustaining function. Thus, the success of the benefactor–beneficiary relationship hinges on the relationship between the (climate) response traits of the benefactor relative to its ability to express the (ameliorating) functional effect trait supporting the beneficiary under a changing environment.

Functional effect traits of a habitat-former are often related to morphology and can be altered by climate change. Known changes in species morphology associated with climate changes include reduced average body size in ectotherms [56]. Likewise, calcifying organisms, including important habitat-formers such as bivalves, may reduce their growth to compensate for increased metabolic costs incurred in acidified seawater [57]. Reduced size may confer weaker ability to deliver benefits to other species. Calcifiers may also experience changes in the chemical makeup of their shells under ocean acidification and warming. This may render them less structurally robust to physical forcing, reducing their ability to serve as anchoring structures for marine diversity [58].

Modifications in phenology may also reduce stress-buffering capacity. For example, on the coasts of British Columbia, experimental warming delayed the development of annual intertidal algae [59], potentially exposing associated species to desiccation and heat stress during spring low tides.

### Viable populations under changing climates

The ability of a habitat-former to maintain viable populations at the edges of its distribution or within warming hotspots determines its potential to act as a critical refugium (Fig 3). In some cases, habitat-former populations have collapsed at the warmer limit of their distribution [60,61]. In other cases, poleward shifts have occurred without changes at the equatorial range edge. For example, reduced risk of winter freezing has promoted poleward migration of some mangroves at the expense of salt marshes but with no significant equatorial edge contraction [62]. In the southern hemisphere, tropical corals and seagrasses have expanded toward higher latitudes without modifying their northernmost boundaries [63,64].

In addition, climate rescuer populations should not undergo thinning during hot seasons or extreme atmospheric events (i.e., exhibit large temporal fluctuations) because they might become too sparse to buffer environmental stress. Since habitat modification is often density dependent [65], assessing whether there is a minimal (threshold) population density or size that is needed for benefits to accrue seems crucial.

## Active management of habitat-formers to mitigate biodiversity loss

By virtue of their potential to ameliorate environmental stress, habitat-former populations could be managed as a tool against climate-driven loss of biodiversity (Fig 3). Major threats to marine habitat-formers and approaches to their conservation have been thoroughly reviewed elsewhere [66] and will not be reiterated here. Instead, we outline a number of actions to sustain habitat-formers facing novel climatic conditions as well as population traits enhancing their effectiveness and reliability in rescuing stress-sensitive species.

### Enhancing habitat-former tolerance to novel climatic conditions

#### Genotype selection

Persistence of target habitat-former populations can be enhanced by selecting stress-tolerant genotypes. Genetic variation in traits relevant under global change seems high in coastal biota [67], and novel quantitative genetic analyses can provide accurate estimates of persistence probability of wild populations [68]. High genetic variation occurs among populations with reduced gene flow but also within the same population. For example, resilience to heatwaves differs between shallow and deep genotypes of the same populations of the seagrass *Posidonia oceanica* [39]. If this is caused by inherited genetic adaptation rather than acclimation to different developmental depths, then assisted relocation of such stress-tolerant genotypes—reared either in the lab or in the field—could rescue declining populations and enhance subpopulation connectivity.

#### Synthetic biology

Although its application in the field of conservation is still in its infancy, synthetic biology is moving fast and may represent a strategic tool under future climates if accompanied by thorough risk-assessment and complying with environmental ethics [69]. Organisms have been genetically modified to enhance their resistance to biotic (e.g., disease) and abiotic (e.g., drought, salinity, heat) stressors, both in terrestrial and marine environments [70,71]. Gene editing of a single habitat-forming species may indirectly enhance the persistence of an entire suite of stress-susceptible species under adverse climates. The molecular basis for tolerance to environmental stress has been identified in key habitat-forming species, such as oysters and corals [72,73]. New genome editing techniques, such as CRISPR/Cas9, may rapidly advance this field.

#### Assisted evolution

Tolerance to stress can be enhanced through human-assisted acceleration of natural processes [74]. Short-term variance in biotic or abiotic pressures is critical to build stress tolerance [75]. For example, rapid fluctuations between benign and severe conditions accelerated adaptation to warming in the marine diatom *Thalassiosira pseudonana*, since population size expansion during favorable periods increased the probability of fixing beneficial mutations [76]. Thus, controlled alternation of high- and low-stress phases in mesocosms—climate incubators—may act as an accelerator for adaptation to climate change, as high-stress phases cause selective mortality of sensitive genotypes, while stress relaxation phases allow surviving genotypes to recover and, possibly, reproduce [77].

#### The microbiome

Microbial symbionts influence host physiology, behavior, and resistance to disease [78]. High genetic diversity and fast generational turnover of symbionts can allow rapid adaptation to novel climatic conditions, potentially raising host fitness [79]. Laboratory thermal selection could expand the temperature tolerance range of the coral-dinoflagellate *Symbiodinium* after approximately 80 asexual generations, corresponding to just two and a half years [80]. Although the mechanisms regulating property transfer from the microbiome to the host (i.e., emergence of stress tolerance at the holobiont level) are yet to be fully understood, assisted microbiome evolution might be a formidable tool for raising habitat-former tolerance to novel climatic conditions.

### Enhancing habitat-former population traits under novel climatic conditions

#### Conservation biology

By drawing on conservation and restoration knowledge, population viability of potential climate rescuers can be actively sustained (Fig 3). Habitat-former population size and resilience can be enhanced by supporting connectivity through protection of source populations, restoration of natural migration corridors, or the creation of new ones [81]. In some cases, managed relocation (or assisted migration) of habitat-formers at strategic sites might enhance connectivity among their populations as well as among populations of beneficiary species. Likewise, herbivore release from predation can result in the overgrazing of habitat-forming macrophytes, and trophic cascade restoration could be necessary to foster their persistence [82].

#### Mitigation of other anthropogenic stressors

Control of local/regional anthropogenic perturbations potentially exacerbating the impact of climate stressors will likely enhance habitat-former population resilience to climate and nonclimate stressors [66,83]. For example, removal of excess nutrients enhances the tolerance of canopy-forming macroalgae to increased temperature [83].

#### Biodiversity

A large body of literature suggests a positive relationship between biodiversity and both resilience and temporal stability [84]. Thus, promoting multispecies assemblages of habitat-formers that are, to some degree, functionally interchangeable, may increase the reliability of their positive effects on other species under changing environmental conditions. In addition, greater microhabitat availability in multispecies assemblages of habitat-formers may enhance the coexistence among beneficiary species and, hence, broaden the number of species sheltered from adverse climatic conditions [84]. When desirable, the formation and maintenance of multispecies assemblages could be pursued through active control of competitively dominant species that would otherwise form monospecific stands or through the seeding of subordinate species. Similar actions could be implemented to enhance genotype diversity, although they would require better understanding of competitive hierarchies between clonal genotypes.

#### Ecoengineering

Maritime infrastructures, off-shore installations, and hard coastal defences (breakwaters, seawalls) significantly change species distribution and ecological connectivity [85]. Ecoengineering designs of artificial habitats including conservation or restoration objectives have the potential to turn these changes into an opportunity to sustain climate rescuer populations by supplying suitable habitats or providing new dispersal routes facilitating their migrations and that of beneficiary species. As previously demonstrated in the fields of restoration and conservation [16,17], engineering man-made structures for sustaining target habitat-forming species would be sufficient for attracting a suite of facultative and obligate associated species and represents, therefore, a cost-effective approach.

#### Non-native species

Where native habitat-formers are lacking, non-native species might be considered as alternative climate rescuers, as they may revitalize functionalities that would be otherwise lost, including the support of diverse communities and the provision of climate refuges. The use of non-native species in conservation is still highly debated, but in extreme cases, they may be the only chance of avoiding massive species loss when key habitat-formers decline due to global and regional human-driven changes (Box 2).

**Box 2. The role of non-native species as climate rescuers**

The view that all non-native species represent a threat to native biodiversity has been challenged on the grounds that some of them cause no harm and can contribute to achieving conservation and restoration goals [86,87].

Climate change is predicted to foster invasions via enhanced propagule dispersal and decreased biotic resistance of native communities [86,88]. In addition, poleward shifts of coastal species have been documented throughout the globe [88]. By virtue of their better adaptation to novel climate conditions, non-native species may be the primary cause of native species decline or local extinction. On the other hand, non-natives may replace natives when they decline as a consequence of other anthropogenic stressors. Although the effects of non-native habitat-formers on marine biodiversity are often complex and variable [89,90], there are examples of non-native species compensating, to some extent, for native habitat-former loss. For example, in areas of Chesapeake Bay where native eelgrass beds have retreated, the macroalga *Gracilaria vermiculophylla* provides suitable habitat for the native blue crab *Callinectes sapidus*, a highly valued recreational and commercial species [91]. Positive effects of non-native habitat-formers can scale up to whole communities and influence ecosystem functioning. For example, long-term bioirrigation by the non-native polychaetes *Marenzelleria* spp. alleviates soft-sediment hypoxia in the Baltic Sea [92]. Likewise, the non-native seaweed *Sargassum muticum* confers benthic assemblages greater resistance to warming and acidification than native macroalgal canopies [93].

Of course, the benefits and risks of using non-native species as climate rescuers do not differ from those already described for restoration or conservation practice [94]. Many aspects of biological invasions, including their perception and management, are still highly controversial [95,96]. By no means do we negate the capacity of non-native species to alter native biodiversity and to impair ecosystem functioning; rather, we suggest that their potential to rescue native species from changing climates should not be discarded a priori but benefits and risks fully evaluated on a case-by-case basis.

## Concluding remarks

Amelioration of physical stress by habitat-formers sustains species persistence in harsh environments [14,15]. This service might become increasingly important under future climates. The potential of habitat-formers to act as climate rescuers relies on their ability to maintain key individual and population traits in the face of climate changes. Likewise, the strength of rescuing effects depends upon source-sink dynamics and the interplay of stabilizing and destabilizing forces regulating the coexistence between the benefactor and the beneficiaries as well as among beneficiaries. Thus, current ability to ameliorate environmental conditions is not sufficient in itself to make a habitat-former a climate rescuer species. Nonetheless, some habitat-forming species display the right individual and population traits (Box 3). Drawing from different ecological and biological disciplines, a series of management actions can sustain the strength and reliability of their climate-rescuing effects. Within a multidisciplinary framework (Fig 3), understanding how biogenic habitats influence evolutionary adaptation of beneficiary species to changing conditions and their ability to track suitable climates should be considered a priority. Developing the concept of sustaining habitat-former populations as a nature-based solution to climate change will likely depend on our ability and willingness to address ethical issues in modern conservation, such as those related to the use of synthetic biology, non-native species, assisted species evolution, and species relocation. Finally, the general features of one or a few species that reduce climate-driven abiotic stress for other species that we describe in coastal systems are likely to be found also in other types of ecosystems. For example, heat tolerance of freshwater gastropods is lowered in hypoxic conditions [97] and may be sustained by macrophyte oxygen production. In high-alpine systems, some cushion plants mitigate the effects of warming on native grasses [9]. Likewise, during drought events, canopy-forming mosses enhance the survival of smaller mosses and hepatics in their understory [98]. Thus, the broad conclusions we derive for coastal ecosystems under climate change may also apply to other ecosystems.

**Box 3. Examples of potential climate rescuers**

**Climate rescuer on the sand**

Sea cucumbers play an important role in coastal environments since they bioturbate sediments and recycle nutrients, sustaining the diversity and functioning of benthic communities [99]. The sea cucumber *Holothuria scabra* (the “sandfish,” Fig 1F) is distributed throughout the Indo-Pacific region, between 30° N and 30° S of latitude. It is an active burrower and enhances sediment oxygenation, buffering negative effects of hypoxia caused by eutrophication and warming [33]. In addition, it can foster seagrass growth and productivity via remineralization of nutrients and/or their release from sediment pore water [99], potentially triggering a facilitation cascade. This species is cultured, and it seems able to rapidly adapt to variable environmental conditions (e.g., salinity, temperature) through behavioral and molecular mechanisms [100,101]. For instance, in aquaculture facilities, extreme water temperatures exceeding 31 °C caused no mortality of juveniles and, indeed, fostered their growth [102]. Finally, the entire mitochondrial genome of this species has been sequenced [103]. For the reasons above, this species may offer a nature-based solution for alleviating the impact of temperature-driven hypoxia.

**Climate rescuer on the rocks**

The brown macroalga *Fucus vesiculosus* (Fig 1C) occupies wide ecological and geographical ranges. Presently, it spans latitudes from above 70° N (Norway) to near 30° N (Morocco), withstanding, at low tide, extreme freezing (e.g., Labrador Sea), extreme heat (e.g., above 40 °C in Iberia), and variable salinities (estuaries, the Baltic Sea). It can function as climate rescuer for taxa beyond the southern limits of most intertidal fucoid seaweeds of the NE Atlantic, which can be vertically compressed and geographically restricted beyond the northwest Iberian climate refugium [104]. In contrast, *F*. *vesiculosus* extends further south, persisting in more extreme conditions. Although it suffered the loss of many populations of a southern genetic lineage [105], reciprocal transplants showed that populations that persisted from this southern lineage have better adaptive traits for their habitat [106]. In this species, the costs of thermal stress to cellular metabolism (recorded as molecular heat shock response) can be escaped when high temperatures co-occur with rapid extreme desiccation [36]. Producing large quantities of recruits of *F*. *vesiculosus* is a standard procedure because this species has been widely used for decades as a model in developmental biology, reproductive ecology, and ecophysiology, including in experimental field outplants [107]. Because the species is easily propagated and the southern populations have the capacity to withstand heat stress and maintain large canopies in areas where few other large intertidal canopies exist, it may offer a nature-based solution for alleviating the impact of multiple stressors on intertidal community diversity and abundance along its warm range limits.
